# Pharmaceutical Analysis of Inpatient Prescriptions: Systematic Observation of Hospital Pharmacists’ Practices in the Early User-Centered Design Phase

**DOI:** 10.2196/65959

**Published:** 2025-04-25

**Authors:** Jesse Butruille, Natalina Cirnat, Mariem Alaoui, Jérôme Saracco, Etienne Cousein, Noémie Chaniaud

**Affiliations:** 1 ENSC – Ecole Nationale Supérieure de Cognitique, Bordeaux INP, 109 Av. Roul, Talence, 33400, France, 33 5 57 00 67 00; 2 PharmIA SAS, Paris, France; 3 IMS – Laboratoire de l’Intégration du Matériau au Système, CNRS UMR5218, Talence, France; 4 ULR 7365 – GRITA, Université de Lille, Lille, France

**Keywords:** human factors, user experience research, user-centered design, professional practice, clinical pharmacy information systems, pharmacy service, systematic observation, pharmaceutical, pharmacy, pharmacist, France, prescription, user-centered, adverse drug events, ADEs, clinical decision support system, CDSS, drug-related problems, patient management, safety, electronic medical record, EMR, pharmaceutical analysis, electronic health, eHealth, hospital

## Abstract

**Background:**

The health care sector’s digital transformation has accelerated, yet adverse drug events continue to rise, posing significant clinical and economic challenges. Clinical decision support systems (CDSSs), particularly those related to medication, are crucial for improving patient care, identifying drug-related problems, and reducing adverse drug events. Hospital pharmacists play a key role in using CDSSs for patient management and safety. Human factors and ergonomics (HFE) methods are essential for designing effective, human-centered CDSSs. HFE involves 3 phases—exploration, design, and evaluation—with exploration being critical yet often overlooked in the literature. For medication-related CDSSs, understanding hospital pharmacists’ tasks and challenges is vital for creating user-centered solutions.

**Objective:**

This study aimed to explore the actual practices and identify the needs of hospital pharmacists analyzing electronic prescriptions. This study focused on the preliminary stage of the user-centered design of a pharmacist-centered CDSS.

**Methods:**

The study involved observing 16 pharmacists across 5 hospitals in mainland France (a university hospital, 2 large general hospitals, a smaller general hospital, and a specialized clinic). Pharmacists were selected regardless of expertise. The observation method—systematic in situ observation with shadowing posture—involved following pharmacists as they analyzed prescriptions. Researchers recorded activities, tools used, verbalizations, behaviors, and interruptions, using an observation grid. Data analysis focused on modeling pharmacists’ cognitive work, categorizing activities by action type, specificity, and information source. Sequential time data analysis and distance matrices were used to generate hierarchical clustering and identify similarity groups among the pharmacists’ analyses. Each group was described using its typical sequences of analysis and related covariates.

**Results:**

In total, 16 pharmacists analyzed and validated electronic prescriptions for 140 patients, averaging 5.48 minutes per patient. They spend 91% of their time searching for information rather than transmitting it. Most information comes from the list of prescriptions, but it is the time spent in electronic medical records (EMRs) that dominates at the heart of the analysis. Pharmaceutical interventions are most frequently transmitted in the last third of the sequence. The pharmaceutical analyses were grouped into 4 clusters: (cluster A, 22%) interventionist clinical analysis with extensive crossing of various sources of information and almost systematic pharmaceutical interventions; (cluster B, 52%) most common clinical analysis focusing on EMRs and biology results; (cluster C, 13%) logistical analysis, focusing on the pharmacy workflow and the medication circuit; and (cluster D, 13%) quick, trivial analyses based exclusively on the list of prescriptions.

**Conclusions:**

The pharmaceutical analysis process is complex and multifaceted. Pharmacists are detectives, accessing a wealth of information to discriminate drug-related problems and respond accordingly. They also carry out different types of analysis, which lead to different needs and require different solutions from CDSSs. This exploratory study is an essential prerequisite for meeting the challenge of designing tools to support pharmaceutical analysis and pharmacists.

## Introduction

### Background

The digital transformation of the health care sector has accelerated over the past decade [1]. This digitization has reduced some risks associated with the use of health care products, such as handwriting-induced confusion [2]. However, adverse drug events continue to represent a significant clinical and economic burden for populations [3]. This paradox is due to the very diverse origins of adverse drug events and to the fact that the computerization of health care organizations corrects some drug-related problems but introduces many others, such as lack of ergonomics and alert fatigue [4,5]. Nowadays, health care providers must make more complex decisions [6], with an exponential increase in clinical and pharmaceutical information. Knowing that certain patient data are available, knowing how to access the data, having the time to search for information, and keeping up to date with the latest treatment recommendations are all challenges faced by professionals in their daily practices [7,8]. Out of this chaos came the interest in clinical decision support systems (CDSSs) to help clinicians make informed decisions and to derive full benefit from hospital information systems [9]. In particular, medication-related CDSSs have the greatest potential to benefit patient care and reduce drug-induced iatrogeny [10-12].

Hospital pharmacists are key users of CDSSs. The pharmacy department is responsible for the entire hospital’s health care product circuit, and the pharmacist’s activity is essential to patient safety and the reduction of health care expenditure [13]. Several studies have demonstrated the value of pharmacist-oriented CDSSs for patient management [10-12]. Of course, in health care, the patient’s well-being is the main concern. Nevertheless, it is also necessary to consider the well-being of health care providers because they are responsible for the majority of medication-related errors [14]. This is why human factors and ergonomics (HFE) methods are fundamental in promoting the human-centered design of systems that support individuals and teams. As defined by Holden et al [15], “HFE is a scientific and practical human-centered discipline that studies and improves human performance in sociotechnical systems.” It is also a standard of practice [16], with applications in many fields: aviation, surface transportation, military, and energy. In this respect, HFE methods are equally applicable to pharmacy challenges and medication-related CDSSs [15,17,18].

Using HFE to design digital products involves three phases [19]. (1) *Exploration* (or *analysis*) aimed at examining users in their natural environment, determining their tasks, activities, goals, constraints, and needs. Here, the real user problems are identified. (2) *Design* takes advantage of exploration data to provide solutions to identified problems. This ranges from simple wireframes to more complete prototypes. (3) *Evaluation* helps to assess ideas and the proposed design in order to optimize and correct it. It involves testing and scoring the user experience, ergonomics, and messages in both form and content.

The first stage is fundamental to the success of the other two. However, HFE studies in the pharmacy sector tend to focus on the design or evaluation phase [20,21]. The same is true of studies on CDSSs [22-24]. Exploring the practices of hospital pharmacists would appear to be a crucial step, particularly for the development of medication-related CDSSs. Software editors need to study the complexity of the task of analyzing and validating electronic prescriptions in order to design products in accordance with the principles of human-centered design [16].

### Prior Work

HFE methodologies are now well established in the health care sector, with a growing number of publications [25]. They all deal with exploration, design, and evaluation, but some phases of human-centered design are better represented than others. Evaluations are clearly the most represented [20,21,23], often involving products already designed or at the end of development, on which usability studies are carried out [26,27]. Unfortunately, these studies give little indication of the earlier stages, unless they also deal with design or co-design [19,22].

In fact, it is rare to find publications that focus solely on the analysis phase, especially in relation to health care providers and pharmacists. Studies focus on the patient [19,28,29] or on the patient’s involvement in the health care system [30]. There are also studies on physicians [31-33], but these tend to focus on the usability of already implemented digital systems [34,35]. Finally, a few studies focus on the importance of the physician-pharmacist relationship for patient safety [12,36]. These studies use questionnaires, interviews, and focus groups, which are HFE methods that have the disadvantage of transcribing user subjectivity rather than actual behavior [37]. More objective methods, such as systematic observations, are better suited to identifying problems and needs for pharmacy research and clinical practice [15].

Several studies use observations as part of their methodology [38-46]. However, they tend to complement other methods in order to diversify data sources and reduce the risk of systematic bias based exclusively on reported behaviors and experiences [38]. Observations are treated here from a qualitative point of view, rather than as quantifying the activity of professionals. Moreover, as with all exploratory publications, the study participants are patients [38,39], physicians [40,41], nurses [42], and health care professionals as a whole [43-45], not just hospital pharmacists. Finally, they are not always user-centered [42], but rather focused on products [45,46] and their usability [39].

Ultimately, some studies have examined pharmacists’ practices using quantitative field observations [47-51]. They do not necessarily focus on HFE methods but rather on the study of professional practice to improve the quality and safety of care [52]. One focuses on community pharmacists and the other 3 on hospital pharmacists. However, they do not deal specifically with the pharmaceutical analysis of prescriptions, but with the work pattern as a whole, and the impact of the medication management system on this work pattern. In the context of developing a CDSS, the information provided by these studies is interesting but does not provide the exploratory data needed to design a prescription analysis support module. Moreover, it is difficult to retrieve exploratory data from the design and evaluation phases, as they only give access to the tip of the iceberg of product design: that is, “What was done?” and not “Why was it done?” To the best of our knowledge, there are no HFE or professional practice publications on prescription analysis by hospital pharmacists. There are only recommendations from professional associations, with useful elements for analyzing and improving practice [53,54].

### Goal of This Study

This exploratory study focuses on the first stage of user-centered design: the phase of in-depth understanding of the user, his or her activity, and the context in which this activity takes place [15]. The aim is to explore hospital pharmacists’ practices when analyzing and validating electronic prescriptions. It also aims to find their specific needs for the design of user-centered CDSSs.

We make several exploratory hypotheses: (1) there are different types of pharmaceutical analysis, (2) pharmacy logistics play a significant role in analysis, and (3) some information is difficult to access for pharmaceutical analysis.

## Methods

### Ethical Considerations

Ethics approval was not sought, as no identifiable health data were collected, and the pharmacists who were observed signed a free and informed consent form after they were informed of their personal data rights, in line with the General Data Protection Regulation (EU 2016/679). The observed pharmacists were not compensated for their participation in this study.

### Recruitment

We visited 5 hospitals in mainland France for a total of 16 pharmacists observed: a university hospital with more than 2000 beds (3 pharmacists), 2 general hospitals with between 1000 and 2000 beds (4 pharmacists), 1 hospital with fewer than 1000 beds (5 pharmacists), and a specialized clinic with fewer than 200 beds (4 pharmacists).

Pharmacists are selected regardless of their expertise or status. This includes pharmacy residents, as they perform much of the validation of hospitalized patients. The only exclusion point is that the pharmacist’s usual task is not to analyze and validate prescriptions (the activity must be performed at least once a week).

The number of participants observed depends on the discrepancy between pharmacists’ practices: the greater the discrepancy, the more observations are needed to describe the different pharmaceutical analysis groups.

### Observations

#### Protocol

Our exploration approach is based on systematic observations in an in situ context [37]. This method enables us to characterize the current state, analyze the environment, and identify the needs of hospital pharmacists. It will also describe how hospital pharmacists access, process, and return information over time in order to analyze and validate patients’ electronic prescriptions.

Each observation consists of following a pharmacist analyzing and validating electronic prescriptions for about an hour. Pharmacists are informed of the objectives of the study before participating. They also receive some instructions, but the full methodological details are not given until the end of the observation to avoid influencing their behavior. Participants are instructed to act as they normally would. They are allowed to verbalize as much as they wish. They can move from one patient to another and can return to a patient who has already been validated. Task interruptions are natural in the professional practice of pharmacists [55], so the participant can interrupt his or her activity at any time.

At the start of each observation, the researcher notes generic information about the environment (date, time, site, and location) and the pharmacist being observed (area of expertise and status). At the end, he or she enters the time and comments, debriefing the participant. In 1 hour of observation, the pharmacist will analyze and validate several electronic prescriptions corresponding to different patients. For each patient, the researcher uses an observation grid to structure data collection.

#### Grid and Posture

An observation grid is completed for each patient (Multimedia Appendix 1). It is organized in the form of a successive list of activities, with each activity necessarily associated with the time required to perform it. Depending on the situation, other information may also be entered: (1) tools used, (2) verbalizations relevant to understanding the activity, (3) behaviors and reactions, and (4) the occurrence of a task interruption and its origin. The researcher records all the pharmacist’s activities, taking care to provide sufficient detail for understanding, but without going too far, at the risk of losing the thread of the process.

The observation method requires the researcher to adopt a posture clearly defined by the study protocol. This is the shadowing technique [56,57]. It consists of following the user’s activities as closely as possible, without intervening directly in the observed situation. However, the researcher may ask the participant questions in order to better understand the reasons for certain behaviors. Here, the researchers are hospital pharmacists familiar with user experience methods. This choice enhances the discernment of very specific professional activities and limits the questions asked of the participant.

### Data Analysis

#### Observation Processing

In order to model pharmacists’ cognitive work from raw observation data, each activity is assigned (1) a type of *action* (search or transmit), (2) a level of *specificity* (generic or specific), and (3) a *source* of information (list of prescriptions, electronic medical records [EMRs], biology results, pharmaceutical interventions, pharmacy logistics, drug information, other information, or alert from prescriptions). Analysis times are represented in relative terms (percentage of total time) rather than absolute values to facilitate the comparison of activity sequences with wide time disparities.

The processing of temporal successions of activities is inspired by the processing of sociological data where populations change state as a function of time (eg, professional status: student → employee → unemployed → employee → retired). In particular, we drew on the work on exploratory analysis in a social context by Mariette et al [58]. To this end, we use the R package *TraMine*R [59] and multidomain case studies (in our case, activities are combinations of 3 domains) [60]. These methods are used to split up activities and generate comprehensible representations.

#### Clustering

Another advantage of these sequential data analysis methods is their ability to generate distance matrices between pharmaceutical analysis sequences [59,60]. This is followed by hierarchical clustering using the R package *cluster* [61] to distribute observations into different similarity groups. Finally, we describe the clusters by matching the graphs of typical sequences for each group (*TraMineR* package), with the description of covariates provided by the R package *FactoMineR* [62] and its class-based modality description function (contingency table, *V* test, and chi-square test).

## Results

### Observations and Sequences of Activities

#### Overview of Observations

The 16 pharmacists analyzed and validated the electronic prescriptions of 140 patients. The distribution of the time data for the 140 observations (mean 5.5; range 0.5-15; median 5, IQR 3.5-7.5 min) was nonnormal (Multimedia Appendix 2).

Pharmacists take an average of 5 minutes and 29 seconds to analyze one patient. The fastest analyses take 30 seconds, and the longest 15 minutes. The time spent on task interruptions is deducted from the total analysis time.

The observations show 17 different types of activity, formed by combining the 3 domains of *action*, *specificity*, and *source* (combinations are available in Multimedia Appendix 3). The representations of the activities (Table 1) separate the action, finesse, and source domains to better compare proportions.

The *action* domain is mostly represented by the search category, accounting for 90.7% of the total analysis time (Table 1). Pharmacists thus spend almost 91% of their time looking for information, and 9% transmitting it. In terms of *specificity*, information is more often generic (66%) than specific (34%). As for the *source* of information, the list of prescriptions is the most common (37.5%), along with the EMRs (28.2%); next in order of frequency are biology results (13.1%), pharmaceutical interventions (7.7%), pharmacy logistics (4.7%), drug information (4%), other information (3%), and alert from prescriptions (1.8%).

**Table 1.** Time distribution of categories in each area (action, specificity, and source). Results are expressed as a percentage of total analysis time.

Area and categories	Time distribution, %
Action	
Search	90.7
Transmit	9.3
Specificity	
Generic	66.0
Specific	34.0
Source	
List of prescriptions	37.5
Electronic medical records	28.2
Biology results	13.1
Pharmaceutical interventions	7.7
Pharmacy logistics	4.7
Drug information	4.0
Other information	3.0
Alert from prescriptions	1.8

#### Time Sequences

**Figure 1.**
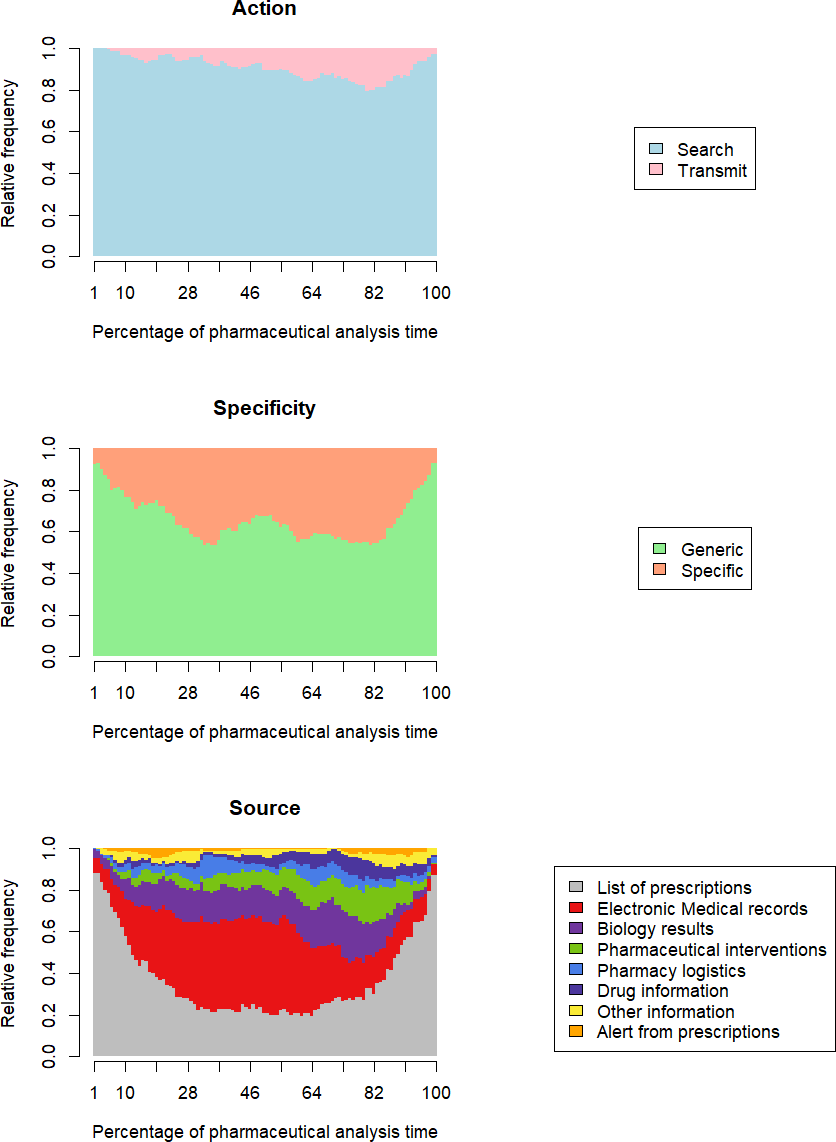
Graphs showing the relative frequency of each type of activity in relation to the percentage of time spent analyzing the prescription.

Time trends in activity categories are shown in the graphs of relative activity frequencies (Figure 1). They are based on graphs of the individual activity sequences of the 140 patients (Multimedia Appendix 4).

For the *action* domain, information search predominates over information transmission throughout the analysis. The transmit category rises slowly from the start of the analysis, peaking at the end, with a frequency of 20% (82% analysis time), before falling back to zero.

For the *specificity* domain, the beginning of the analysis mainly concerns generic information, with a gradual increase in the proportion of specific information as the analysis progresses. After 30% of the analysis time, the share of specific information stabilizes at around 40% of the total information consulted or transmitted. At the end of the analysis, pharmacists return to a large majority of generic information.

For the *source* domain, the list of prescriptions is consulted frequently throughout the analysis (basal frequency 20%), with 2 peaks at the beginning and end. Between 28% and 64% of the analysis time, the EMR is in the majority, representing around 40% of all activities performed during this period. The proportion of “biology results” remains fairly constant, as do “pharmacy logistics,” “other information,” and “alerts from prescriptions.” Pharmaceutical interventions and drug information are poorly represented at the outset but increase to reach a peak of around 82% of analysis time. Details of the relative frequencies of the *source* domain for each observation are available in Multimedia Appendix 5.

### Types of Pharmaceutical Analysis

The distance matrix between analysis sequences is generated by assigning greater weight to the source domain than to the action and specificity domains (ratio of 2:1). The reason behind this ratio is that the source domain is more objective than the specificity domain (less bias) and more discriminating than the action domain (improving the strength of the classification structure). The agglomeration coefficient of the hierarchical clustering is 0.88 (see dendrogram in Multimedia Appendix 6). In order to facilitate interpretation and maximize class coherence, the clustering is divided into 4 groups (A, B, C, and D).

**Figure 2.**
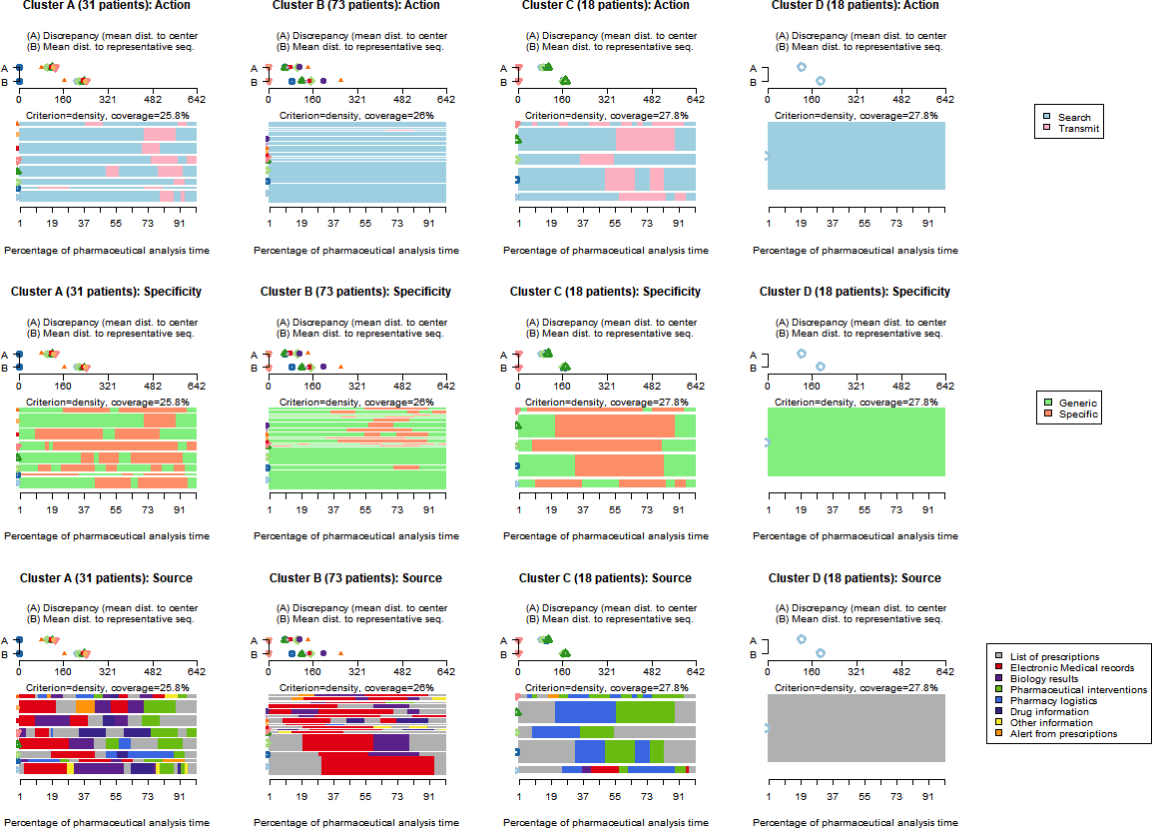
Reference activity sequences for each cluster and domain. Dist.: statistical distance (used to assess the dissimilarity or difference between two statistical objects).

The characterization of pharmaceutical analysis typologies is done using graphs of the reference sequences for each sequence cluster (Figure 2). The results in this figure can be read using the tables describing the clusters according to covariates (Tables2  3).

**Table 2.** Description of each cluster by qualitative variables. Chi-square test *P* value: ***P<.001; **P<.01; *P<.05.

Qualitative variables=Modality	Cluster/modality (%)	Modality/cluster (%)	Global (%)	*V* test
Cluster A (31 patients)				
Validation=With reservations	41.8	74.2	39.3	+4.4***
Status=Associate practitioner	37.5	67.7	40.0	+3.5***
Pharmacist=OBS08	55.6	16.1	6.4	+2.2*
Environment=Shared office	26.7	87.1	72.1	+2.1*
Patient=PAT002	43.8	22.6	11.4	+2.0*
Observation=OBS04	0.0	0.0	8.6	−2.0*
Status=Senior	12.9	29.0	50.0	−2.6**
Department=Medical	11.5	22.6	43.6	−2.7**
Center=CEN04	5.6	6.5	25.7	−2.9**
Validation=Yes	9.1	22.6	55.0	−4.1***
Cluster B (73 patients)				
Center=CEN04	86.1	42.5	25.7	+4.8***
Validation=Yes	70.1	74.0	55.0	+4.7***
Environment=Individual office	82.8	32.9	20.7	+3.7***
Status=Junior	92.9	17.8	10.0	+3.3**
Pharmacist=OBS09	92.9	17.8	10.0	+3.3**
Department=Medical	67.2	56.2	43.6	+3.1**
Pharmacist=OBS11	81.8	12.3	7.9	+2.0*
Patient=PAT002	25.0	5.5	11.4	−2.3*
Center=CEN02	16.7	2.7	8.6	−2.5*
Observation=OBS05	16.7	2.7	8.6	−2.5*
Department=Surgery	28.9	15.1	27.1	−3.3***
Environment=Shared office	42.6	58.9	72.1	−3.7***
Pharmacist=OBS04	0.0	0.0	8.6	−3.9***
Validation=With reservations	23.6	17.8	39.3	−5.5***
Cluster C (18 patients)				
Pharmacist=OBS04	83.3	55.6	8.6	+5.9***
Status=Senior	25.7	100	50.0	+4.9***
Validation=With reservations	29.1	88.9	39.3	+4.5***
Center=CEN01	29.7	61.1	26.4	+3.3**
Environment=Shared office	17.8	100	72.1	+3.1**
Department=Surgery	26.3	55.6	27.1	+2.7**
Center=CEN04	2.8	5.6	25.7	−2.2*
Environment=Individual office	0.0	0.0	20.7	−2.5*
Validation=Yes	2.6	11.1	55.0	−4.0***
Status=Associate practitioner	0.0	0.0	40.0	−4.1***
Cluster D (18 patients)				
Environment=Open space	40.0	22.2	7.1	+2.2*
Pharmacist=OBS06	40.0	22.2	7.1	+2.2*
Validation=Yes	18.2	77.8	55.0	+2.1*
Validation=With reservations	5.5	16.7	39.3	−2.1*

**Table 3.** Description of each cluster by quantitative variables. Chi-square test *P* value: **P<.001; *P<.01.

Quantitative variables	Mean in cluster	Overall mean	SD in cluster	Overall SD	*V* test
Cluster A (31 patients)					
Analysis time (minutes)	7	5.5	2.7	3	+3.2*
Cluster B (73 patients)					
Null	N/A^a^	N/A	N/A	N/A	N/A
Cluster C (18 patients)					
Null	N/A	N/A	N/A	N/A	N/A
Cluster D (18 patients)					
Analysis time (minutes)	2.8	5.5	2	3	−4.1**

a N/A: not applicable.

Cluster A comprises 31 (22.1%) sequences of the 140 patients analyzed. The typical group A sequence includes a wide variety of information *sources*, with many alternating between them. The level of *specificity* is balanced between generic and specific information. The *action* of transmitting occurs mainly at the end of the analysis, in correlation with the “pharmaceutical interventions” *source*. Cluster A analyses took significantly longer for pharmacists. They are mostly carried out by assistant pharmacists, and their validations are mostly made with reservations (pharmaceutical interventions).

Cluster B involves the most patients, with 73 (52.1%) sequences of the 140 patients analyzed. The typical cluster B sequence is characterized by the *action* of searching for information (no transmission), and the *specificity* of the information is more generic than specific. The diversity of information *sources* is lower than in cluster A: pharmacists alternate between the list of prescriptions and the EMRs, with a few passages in biology results and drug information. Cluster B sequences contain almost all the sequences produced by junior pharmacists and give rise to unreserved validations (no pharmaceutical interventions). Cluster B patients are more likely to be hospitalized in medical departments than in surgical ones.

Cluster C contains 18 (12.9%) sequences of the 140 patients analyzed. The typical cluster C sequence devotes most of its analysis time to the *action* of transmitting. Most of the information is specific, with generic information used mainly at the beginning and end of the analysis. Activity *sources* are few when compared to cluster A, and alternate between list of prescriptions, pharmacy logistics, and pharmaceutical interventions. The sequences in cluster C relate more to the analysis practices of senior hospital practitioners who validate surgical services. Validation is generally carried out with reservations (pharmaceutical interventions).

Cluster D also comprises 18 (12.9%) sequences of the 140 patients analyzed. The typical cluster D sequence is the simplest of the 4 groups. It consists solely of search *actions*, with generic *specificity* and the list of prescriptions as its *source*. Cluster D analyses take significantly less time for pharmacists and result in unreserved validation (no pharmaceutical interventions). This type of analysis is more common among pharmacists working in open spaces.

## Discussion

### An Investigative Posture

The goal of this study is to understand how pharmacists analyze prescriptions, and in particular, whether analysis profiles exist. The 16 pharmacists who participated in the observations took an average of 5 minutes and 29 seconds to analyze and validate a patient. Although this value is close to the median (5 minutes), it should be noted that the total range of validation times is rather wide (between 30 seconds and 15 minutes) and that the distribution of values does not follow a normal distribution (Multimedia Appendix 2). We can therefore expect major disparities in analysis typologies, but we will come back to this point later.

From an overall point of view, the pharmacists’ posture is overwhelmingly focused on seeking information from prescriptions (Table 1). Whether they are identifying a drug-related problem, or justifying the absence of a problem, the professionals are constantly questioning the list of prescriptions (Figure 1). A new question leads to a new search, which in turn may lead to further questions. The pharmacists keep in mind (or physically note) the questions that come to mind when reading the list of prescriptions and then access the appropriate source of information. At the end of the analysis, as they return to the prescriptions for final validation, they make a final review in order to “not miss anything important.” Overall, pharmacists are naturally at ease when interacting with the drug list, and it is during these periods that participants exchange the most with the researchers.

In terms of specificity, the results are less clear-cut than for the “action” domain. Specificity depends more on the nature of the information researched or transmitted by pharmacists. Information is more often generic in the list of prescriptions and EMR sources, and predominantly specific in the biology results, pharmaceutical interventions, and pharmacy logistics (Figure 2). The need for specific data tends to come at a more advanced stage in the pharmacist’s cognitive process. In general, a specific activity follows generic research of information and is more likely to take place in a structured data source such as biology results. In the case of generic activities, we find structured information sources (prescription lists) and unstructured sources (EMRs). So, it is not the structure of the information that counts here but rather the fact of having a global view of a situation. Generic searches are therefore a way for pharmacists to retrieve as much information as possible about the drug and clinical context, and then focus on more specific activities.

### Importance of Clinical Contextualization

Searching for information in a patient’s medical record is by far the most cognitively demanding activity for professionals. As soon as participants immerse themselves in the clinical data, their verbalizations diminish, and their posture shifts toward reading and concentration. While the total time allocated to clinical contextualization is the second highest of all analyses (Table 1), it dominates between 20% and 70% of analysis time (Figure 1). Moreover, this activity is expressed as the most time-consuming by the participants and is probably the most critical point of the analysis. Pharmacists find themselves highly dependent on information provided by other medical professionals. The search for clinical data is made all the more difficult by the wide disparities in information architecture from one department to another and from one facility to another. Typically, pharmacists find less relevant information on treatments in surgical departments than in medical specialty departments. As for establishments, the way in which they search for clinical data depends on the software that is used for “electronic medical records” and its affinity with pharmacy practice. Finally, if we compare clinical record activities with biology activities, it is clear that the biology laboratory provides much more structured information, enabling pharmacists to target specific data and waste less time searching for it (Table 1). In short, the lack of structure in clinical data requires hospital pharmacists to make a more intense cognitive effort at the core of their analysis. This validates the hypothesis that access to relevant information is often difficult.

The use of other information sources is more sporadic but no less interesting. The search for drug information is correlated with the transmission of pharmaceutical interventions. Pharmacists have a strong need to verify and justify their transmission actions: Acting as drug experts who inspect prescribers’ actions, they do not want to leave any room for uncertainty. In addition, they have a preventive posture toward their medical colleagues, and many participants double their written intervention with a call to ensure that it is properly transmitted. Curiously, iatrogenic alerts prompted by EMRs are rarely consulted, even though they are presented by vendors as aids to analysis and decision-making. When questioned on this subject, all the pharmacists answered that “these alerts are not appropriate,” as they are “too general” and “irrelevant to the context.” Finally, it is the activities related to pharmacy logistics that are the most difficult to describe from raw observations. A review by typology would be necessary to better understand the place of pharmacy logistics, as well as the disparities between the other analyses.

### Several Types of Analysis

**Figure 3.**
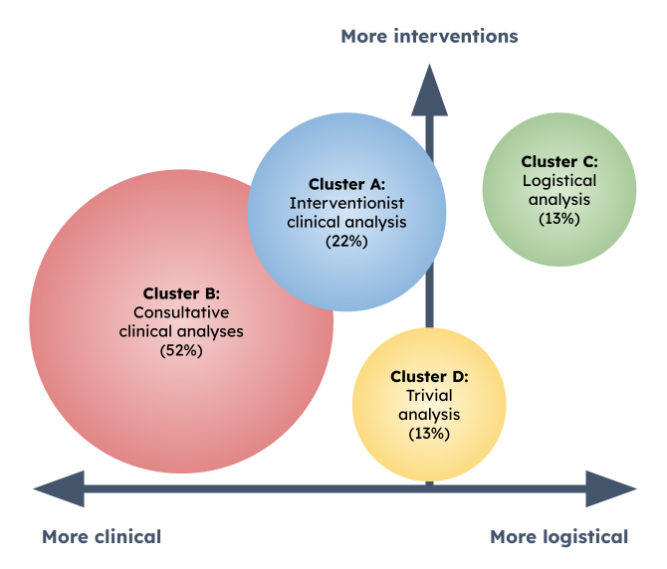
Representation of the 4 pharmaceutical analysis clusters, according to their clinical or logistical trend, and their composition in pharmaceutical interventions. The size of the circles is proportional to the number of analyses concerned.

The classification of analysis sequences into 4 clusters highlights very different analysis typologies (Figure 2). This confirms the first hypothesis of the study. Groups A and B are referred to as “clinical,” as they comprise sequences containing more information from the clinical and biological file than the other clusters. In total, these “clinical” sequences account for almost 75% of the analyses carried out. The remaining 25% is divided equally between clusters C and D. Although cluster D provides fewer elements for understanding professional processes (very rapid analysis, based only on prescriptions), it nonetheless illustrates situations that do not require in-depth expertise and the intervention of a pharmacist. Cluster D is ultimately a group of “trivial” analyses for drug experts. On the other hand, cluster C involves sequences that call on the logistical skills of hospital pharmacists, including their ability to intervene finely in the medication circuit. In the following, cluster C is referred to as the “logistical” group. The terms “clinical,” “logistical,” and “trivial” are introduced to qualify clusters more effectively and to give them a distinctive color. Analysis groups cannot be reduced to their names, as a clinical typology may include a logistical component, and vice versa. The following diagram provides a visual representation of the 4 analysis clusters (Figure 3).

The 2 “clinical” clusters differ mainly in the overall dynamics of their analysis sequences. Cluster A is richer in sources of information, the data sought are more specific, and the sequence of activities is long and complex. Furthermore, these sequences regularly identify drug-related problems and generally lead to validations with reservations (pharmaceutical interventions at the end of the analysis). The value of pharmaceutical analysis of cluster A prescriptions is therefore indisputable, particularly in the fight against iatrogenesis.

Cluster A can be described as “clinical and interventionist,” as opposed to cluster B, which is more “clinical and consultative.” Cluster B’s analysis sequences focus primarily on consulting the patient’s medical file, with the addition of a few biological elements or information on treatments. It is hard to say whether consultative sequences require more cognitive effort from pharmacists than interventionist sequences. In any case, cluster B analyses concern more than 7 out of 10 “clinical” sequences and generally result in full validation (74% of cases). The impact of the clinical advisory analysis is consequently more limited. Finally, it should be noted that cluster B is devoid of logistical aspects, unlike cluster A. This is certainly because cluster A comprises a majority of associate practitioners, who are often more involved in logistics than juniors. In addition, type A analyses are generally more complex, both clinically and logistically, with an important alternation in the specificity and source of information.

The “logistics” cluster includes sequences produced exclusively by hospital practitioners (senior pharmacists). It reflects the increasing specialization of these professionals in the analysis and validation of prescriptions. In fact, their intention is to focus on the medication circuit rather than on iatrogenicity. Their interactions are therefore less with prescribers than with nurses, pharmacy assistants, and automated dispensing systems (dispensing robots and secure cabinets). The clinical aspect, while not completely omitted, tends to be secondary in this type of sequence. As expected, pharmacy logistics therefore play an integral role in prescription analysis.

Finally, “trivial” analyses are not linked to a particular category of pharmacist or hospital. Rather, they appear in relation to clinical situations that do not require the intervention of a pharmacist. This might be a straightforward surgical protocol, a prescription with a limited number of drugs, or standardized palliative care management. In all these situations, pharmacists quickly realize that there are no drug-related problems. However, before opening the prescription list, pharmacists have no way of knowing whether these are “trivial” prescriptions. Given that it represents 13% of analyzed prescriptions, this raises the question of the place of this kind of analysis in the pharmacists’ workflow.

### Identifying Pharmacists' Needs

The diversity of pharmaceutical analysis typologies adds a further challenge to the design of pharmacist-centered decision support systems. Between “clinical,” “logistical,” and “trivial” validation intentions, the needs seem very different, and perhaps these intentions should be treated separately.

For “clinical” analysis, contextualization is a key concern. Pharmaceutical professionals need rapid access to relevant information, that is, without having to search through all the documents and unstructured data. Further, in the case of interventionist analyses, additional elements need to be included to facilitate the drafting and justification of pharmaceutical interventions.

For “logistics” validations, the need lies in the integration of stock management software and the import of highly specialized information (stock-outs, equivalences, and traceability). Since the act of validating a prescription means that it has been approved by the pharmacist, whatever the type of analysis performed, it is important that “logistical” analyses include some support for “clinical” analysis. This is to ensure that iatrogenic risk is minimized.

For “trivial” validations, the need for a decision-support system is more for prioritization. In fact, the time pharmacists devote to analysis is precious and should be used in priority for other types of validation.

### Limitations

One of the first limitations of observation methods is their representativity [52]. In fact, this method requires a great deal of resources to collect field data and then to process and analyze the activities. This limits the number of observations made and calls into question the generalizability of the results. This is why the study of pharmaceutical analysis typologies is based on the analysis sequences of each patient (140 in all) rather than on each pharmacist (16 in all), and why the link between the analysis clusters and the pharmacists observed is not discussed. This also has to do with intraindividual variability: in the study, there was no indication of whether pharmacists changed their testing intention according to the time of day, the day of the week, or the month of the year. It is simply a 1-hour observation of their pharmacy practice at a given point in time.

Although our study includes 5 different hospitals, it would be necessary to generalize the results to other French hospitals and regions. Based on our data, it is not possible to assess the extent of variability attributable to different hospital workflows or pharmacist profiles. Indeed, it would be valuable to analyze the impact of hospital regions, facilities with varying workflows, or pharmacists with differing levels of experience on hospital pharmacy practices.

The aim of the study was to create a general model of pharmaceutical analysis and to form analysis groups. Unfortunately, by generalizing, it is not possible to detail every action performed by professionals. Moreover, in the health care environment, actions are rarely complete and linear [52], making it impractical to group very precise tasks into 140 different sequences. It is also for this reason that task interruption data were not exploited, as there were too many hazards and their use was not relevant to the study’s objective.

### Comparison With Prior Work

Few publications deal with the analysis of hospital pharmacists’ practices and needs with regard to the pharmaceutical analysis of prescriptions. And most of these are usability tests rather than exploratory studies. Nevertheless, 4 studies have similar methodologies and subjects of observation [47-50]. They all use a pay-for computerized data collection tool, the validated Work Observation Method By Activity Timing (WOMBAT, Macquarie University) software [52]. This tool is very useful for observing complex situations, enabling the collection of multiple dimensions of work (who, what, when, why, and how). However, our situation is specific to a given practice, that is, analyzing prescriptions. In the publications cited above, the perception of tasks is more global, more general, and more exhaustive of the daily work of pharmacists. By combining the *action*, *specificity*, and *source* domains, we are able to better characterize tasks relating specifically to pharmaceutical analysis. On the other hand, we lose information on social interactions and other tasks such as dispensing and medication reconciliation. This difference in objectives explains why it is difficult to compare the times obtained in our study with those of other publications: the tasks studied are not homogeneous.

Three of the studies use observations to make comparisons. These are either before-and-after comparisons of the computerization of the prescription management system [50], sometimes adding a comparison between different countries [48], or comparing the activities of pharmacists with those of pharmacy technicians [47]. None of these studies attempt to group together or identify specific typologies of professional practice; the methodologies are based solely on descriptive statistics. Further, the observations are carried out in just 1 or 2 hospitals, with observed pharmacists working in the same ecosystem. This makes it more difficult to eliminate the bias associated with environmental conditions and to distinguish the needs associated with each type of pharmaceutical practice [49]. These studies are therefore not really to be considered as first steps in user-centered design.

Finally, there are some very interesting points of agreement between prior research and our results. Hospital pharmacists spend the majority of their time reviewing prescriptions, affirming the importance of the analysis activity in professional practice. We can also see from the publication by Lo et al [50] that the task of reading the patient’s medical record ranks second in frequency, just after reviewing the prescription list, regardless of the presence or absence of an electronic medication management system. Prior works thus confirm the importance of clinical contextualization in making medicines safer. Even if it is difficult to compare our study to prior work, the problems observed seem to be common. We have every reason to believe that pharmaceutical analysis is an activity whose workings do not differ fundamentally from one organization to another, and whose same needs persist over time. It should also be noted that we do not know if these needs are well addressed by CDSSs. It would be interesting to make this assessment in a future study, comparing it with the needs identified in our exploratory study.

### Conclusions

This exploratory study focuses on pharmaceutical analysis and validation practices for intrahospital prescriptions. We can confirm that the process of analyzing is multifaceted and complex. It depends on the clinical or biological situation, the treatments involved, and the profile of the validating pharmacist. Given that all patients must benefit from the same quality of care, software product teams need to ensure that user needs coincide with the regulatory and ethical constraints of the health care sector. They must also take into account the diversity of pharmaceutical analyses and their specific needs. User-centered design solutions can emerge from this type of exploratory study, leaving designers free to define the right challenges to address and draw innovative ideas from them.

Beyond the interest of this study for the development of useful, usable, and acceptable digital solutions, the work presented here proposes a singular methodology for evaluating the activity of hospital pharmacists. We hope that this methodological contribution will provide a better understanding of the complexity of this medicotechnical profession and make it easier to measure professional practice evolutions, especially after the introduction of a pharmacist-oriented CDSS.

## Supplementary material

10.2196/65959 Multimedia Appendix 1 Observation grid for a prescription or patient.

10.2196/65959 Multimedia Appendix 2 Histogram of total analysis time versus number of patients. The horizontal whisker box represents quartiles, median, and limit values. The mean time is represented by the vertical line.

10.2196/65959 Multimedia Appendix 3 Different types of activity observed. They are formed by combining the categories of action, specificity, and source. The description column provides a better understanding of the activities resulting from these combinations.

10.2196/65959 Multimedia Appendix 4 Sequences of activities in each domain for the 140 patients analyzed. The abscissa represents the percentage of prescription analysis time, and the ordinate corresponds to the patient index.

10.2196/65959 Multimedia Appendix 5 Relative frequencies of source domain activities for each of the 16 observations (pharmacists).

10.2196/65959 Multimedia Appendix 6 Reference activity sequences for each cluster and domain.

## Abbreviations

CDSS: clinical decision support system
EMR: electronic medical record
HFE: human factors and ergonomics
WOMBAT: Work Observation Method by Activity Timing
