# Supplementary figures and images for: Pharmaceutical Analysis of Inpatient Prescriptions: Systematic Observation of Hospital Pharmacists’ Practices in the Early User-Centered Design Phase
Source: JMIR Hum Factors. 2025 Apr 25;12:e65959. doi: 10.2196/65959 (PMC12048037; doi:10.2196/65959)

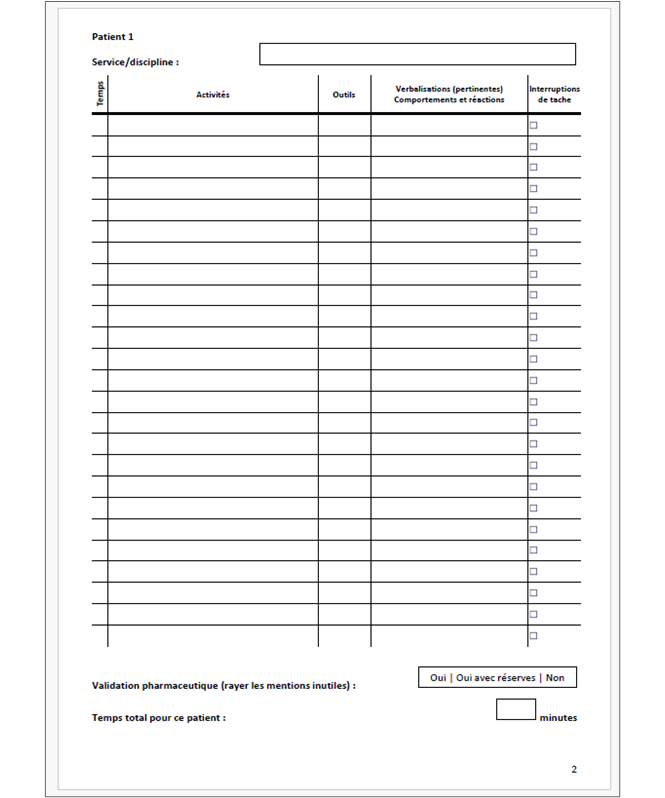

Supplement: Multimedia Appendix 1 [file humanfactors-v12-e65959-s001.png]

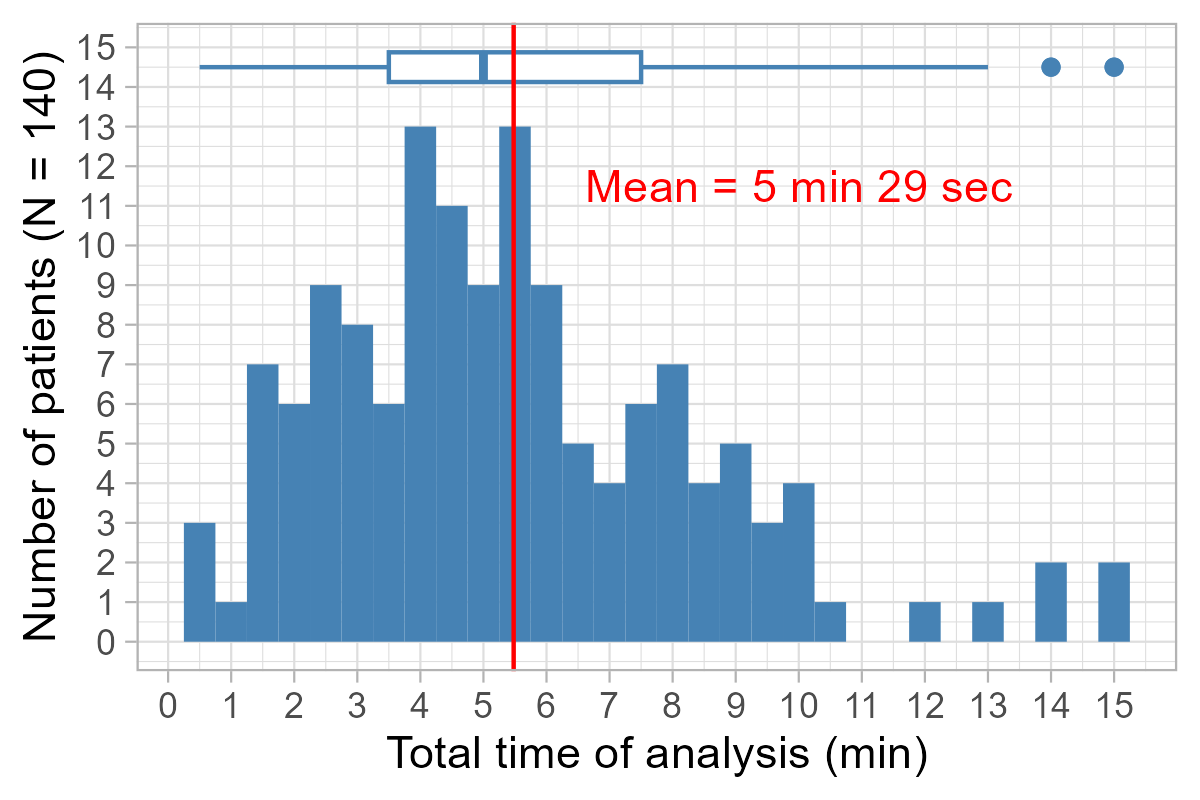

Supplement: Multimedia Appendix 2 [file humanfactors-v12-e65959-s002.png]

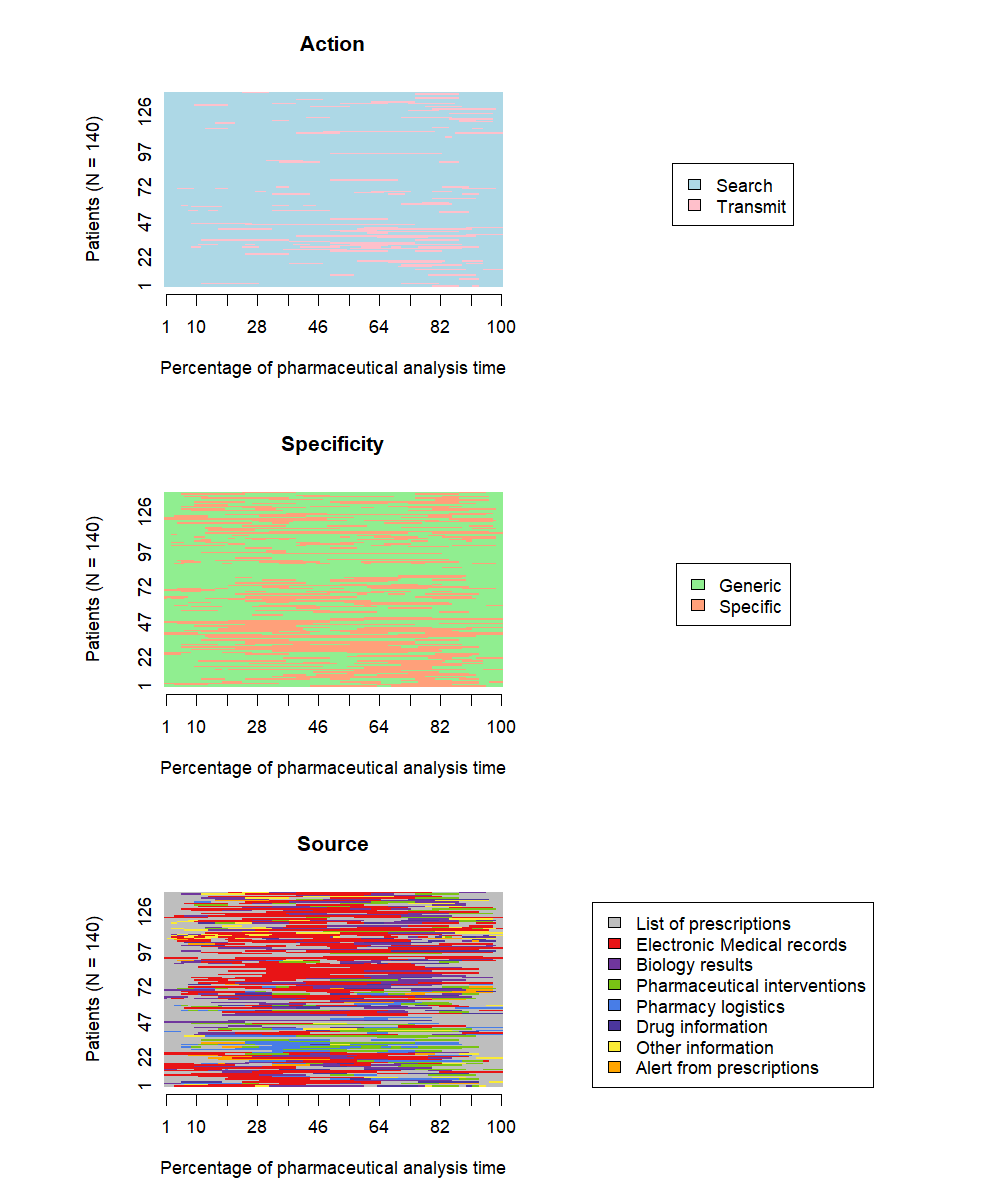

Supplement: Multimedia Appendix 4 [file humanfactors-v12-e65959-s004.png]

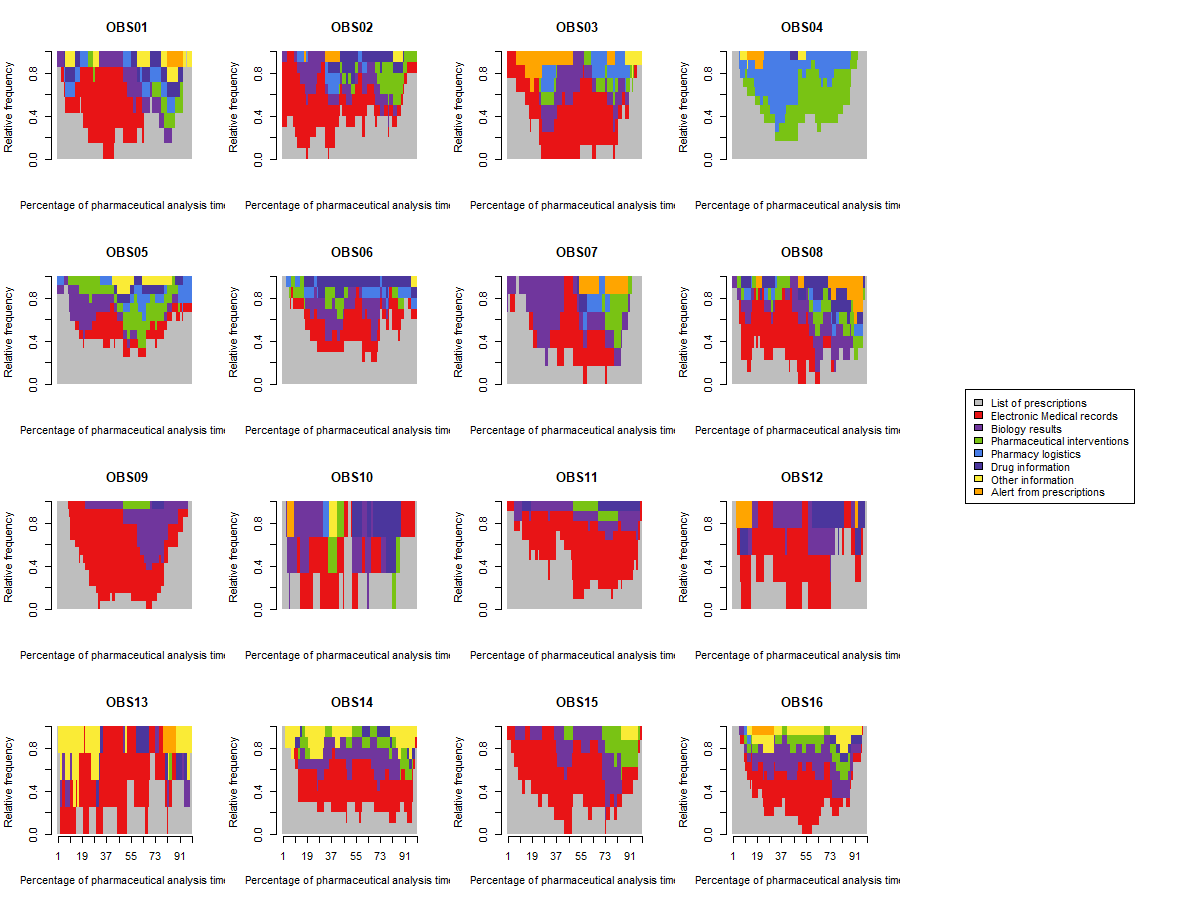

Supplement: Multimedia Appendix 5 [file humanfactors-v12-e65959-s005.png]

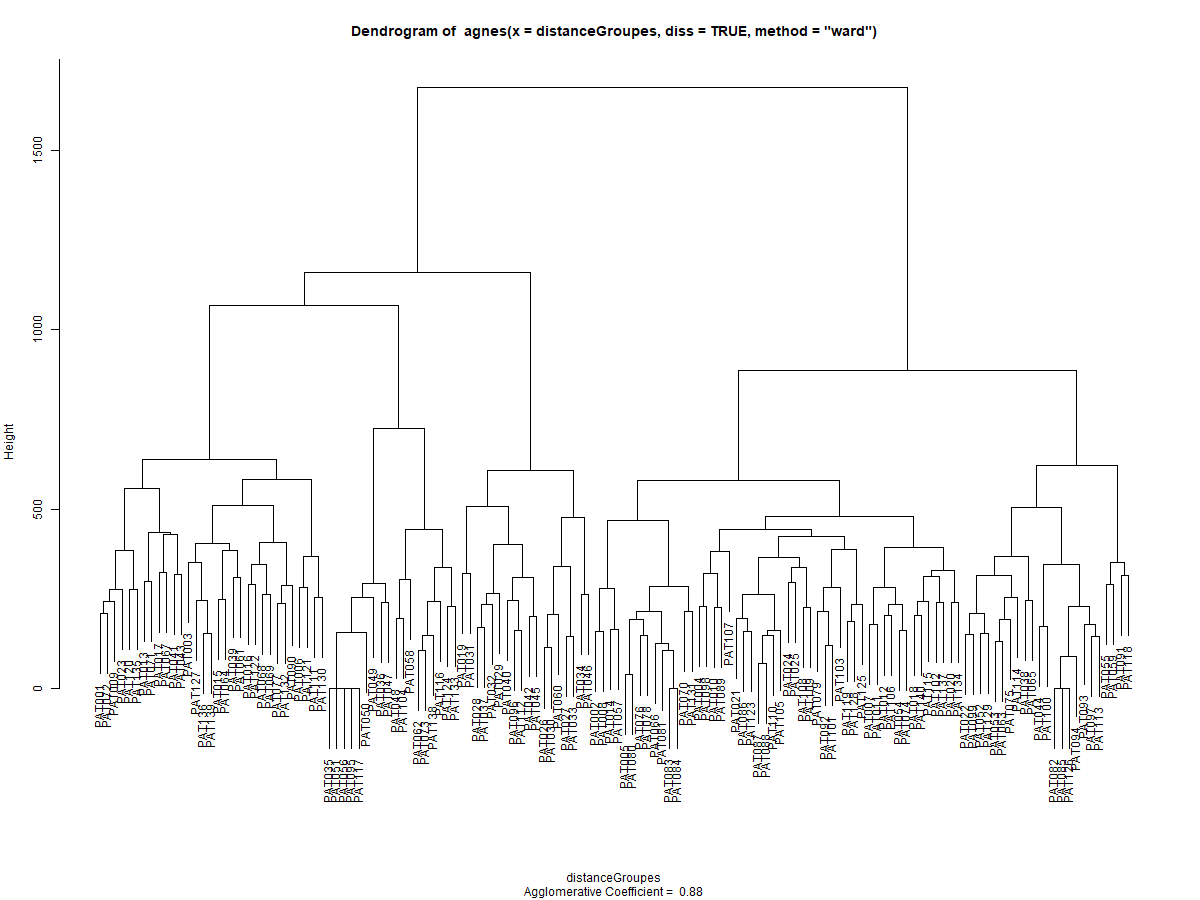

Supplement: Multimedia Appendix 6 [file humanfactors-v12-e65959-s006.png]
