# Supplementary material for: Pharmaceutical Analysis of Inpatient Prescriptions: Systematic Observation of Hospital Pharmacists’ Practices in the Early User-Centered Design Phase
Source: JMIR Hum Factors. 2025 Apr 25;12:e65959. doi: 10.2196/65959 (PMC12048037; doi:10.2196/65959)
Supplement: Multimedia Appendix 3 [file humanfactors-v12-e65959-s003.docx]

| **Action** | **Specificity** | **Source** | **Description** |
| --- | --- | --- | --- |
| Search | Generic | List of prescriptions | Learn about prescriptions as a whole |
| Search | Specific | List of prescriptions | Learn about a specific prescription line |
| Search | Generic | Electronic Medical records | Read the overall medical record |
| Search | Specific | Electronic Medical records | Take note of a specific component of the medical record |
| Search | Generic | Biology results | Review biological parameters |
| Search | Specific | Biology results | Learn about specific biological parameters |
| Transmit | Specific | Pharmaceutical interventions | Transmit a pharmaceutical intervention (written or oral) |
| Search | Generic | Pharmacy logistics | Read generic logistics information (inventory or pharmacy settings) |
| Search | Specific | Pharmacy logistics | Receive specific logistical information (prescription line) including logistics alerts |
| Transmit | Specific | Pharmacy logistics | Transmit logistics (settings and dispensing) |
| Search | Generic | Drug informations | Collect generic information on treatment use (mostly Physician's Desk Reference) |
| Search | Specific | Drug informations | Collect specific information on the use of a treatment (especially expert databases) |
| Search | Generic | Other informations | Collect information (notebook) for working memory (draft) |
| Search | Specific | Other informations | Collecting information (notebook) for a specific purpose |
| Transmit | Specific | Other informations | Transmit information in patient records or prescriptions |
| Search | Generic | Alert from prescriptions | Review alerts |
| Search | Specific | Alert from prescriptions | Learn about a specific alert (excluding logistics alerts) |
